# Potential Applications of Zeolite Membranes in Reaction Coupling Separation Processes

**DOI:** 10.3390/ma5112101

**Published:** 2012-10-30

**Authors:** Michael O. Daramola, Elizabeth F. Aransiola, Tunde V. Ojumu

**Affiliations:** 1 Biochemical and Reactions Engineering Group, Department of Chemical Engineering, Obafemi Awolowo University, Ile-Ife 220005, Osun State, Nigeria; E-Mail: aransiola4@yahoo.com; 2 Department of Chemical Engineering, Cape Peninsula University of Technology, Cape Town 8000, South Africa; E-Mail: ojumut@cput.ac.za

**Keywords:** zeolite materials, applications of zeolites, zeolite membrane reactors, process intensification

## Abstract

Future production of chemicals (e.g., fine and specialty chemicals) in industry is faced with the challenge of limited material and energy resources. However, process intensification might play a significant role in alleviating this problem. A vision of process intensification through multifunctional reactors has stimulated research on membrane-based reactive separation processes, in which membrane separation and catalytic reaction occur simultaneously in one unit. These processes are rather attractive applications because they are potentially compact, less capital intensive, and have lower processing costs than traditional processes. Therefore this review discusses the progress and potential applications that have occurred in the field of zeolite membrane reactors during the last few years. The aim of this article is to update researchers in the field of process intensification and also provoke their thoughts on further research efforts to explore and exploit the potential applications of zeolite membrane reactors in industry. Further evaluation of this technology for industrial acceptability is essential in this regard. Therefore, studies such as techno-economical feasibility, optimization and scale-up are of the utmost importance.

## 1. Introduction

**Figure 1:**
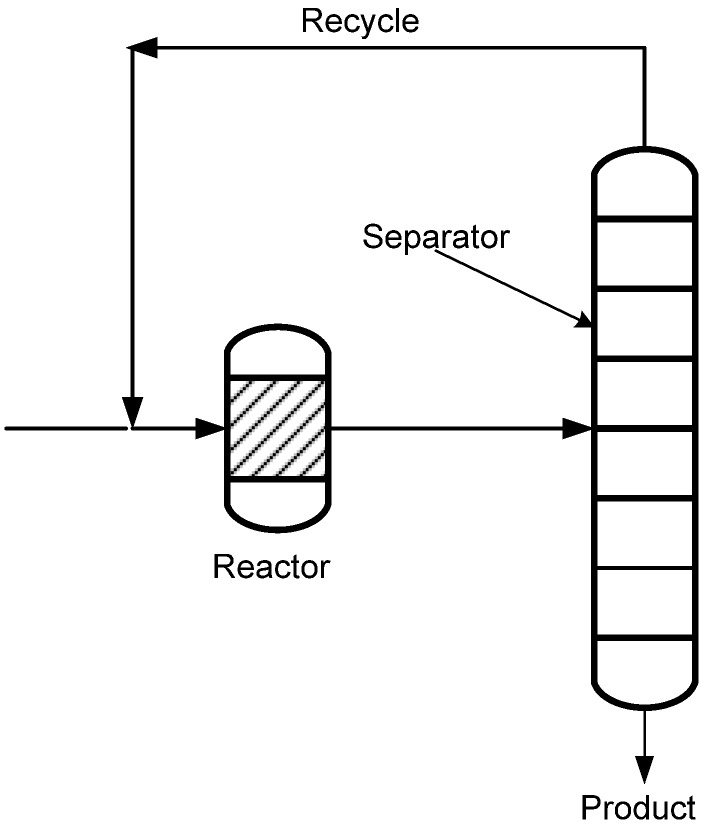
Conventional reaction followed by separation in chemical industry (adapted from reference [2]).

Globally, energy efficiency and energy saving are important components of government policies in response to a range of challenges that include perceptions of resource scarcity, high energy prices, security of energy supply and environmental protection. Consequently, policies in the chemical industry are formulated in-line with the government policies to improve on energy efficiency and energy saving of the industry. Traditionally in the chemical industry, a chemical process consists of a reaction unit followed by a separation unit (see Figure 1). In the reaction unit conversion of reactants to desired and undesired products occurs and removal of the desired product from the reaction mixture takes place in the separation unit. In this conventional system, a huge amount of energy is consumed translating into enormous operating costs. In 2006, the total world energy consumption was 495.6 quintillion Joule (J) and the industrial sector accounted for about one-half of the total world energy consumption [1]. For instance, in the petrochemical industry, energy accounts for more than 60% of the industry’s cost structure. As an example, in 2006, five industries accounted for about 68% of the total energy consumed in the industrial sector while the chemical sector is the largest industrial consumer of energy with about 29% of the energy [1]. Despite the current economic downturn, it is expected that the world energy consumption will increase up to 711.9 quintillion Joules (J) over the 2006 to 2030 period due to the expected growth of the world’s real Gross Domestic Product (GDP) on purchasing power parity averaged at 3.5 percent annually [1]. Over the next 25 years, worldwide industrial energy consumption is expected to grow from 183.8 quintillion Joule (J) in 2006 to 257.9 quintillion Joule (J) in 2030 at an average annual rate of 1.4% [1].However, more energy-efficient technologies in the chemical industry could contribute significantly to nationwide and worldwide energy savings and a reduction of CO_2_ emissions.

**Figure 2:**
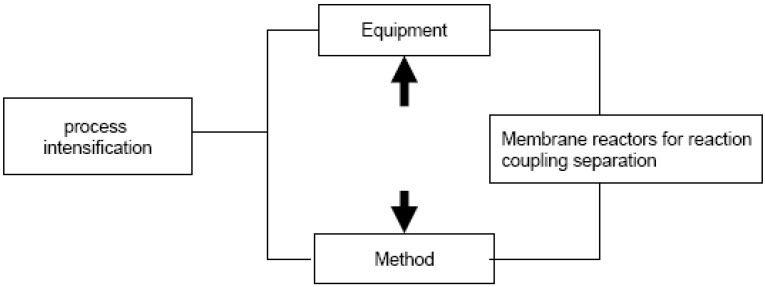
Process intensification and its components for membrane reactor concept for the reaction coupling separation process (adapted from Reference [3]).

Process intensification of a chemical process can provide a way to alleviate this problem. From an engineering point of view, the vision of process intensification through multifunctional reactors has activated research on membrane reactors. A concept of process intensification for the chemical industry involving the use of membrane reactors is depicted in Figure 2. In a chemical process, process intensification can be done on method or equipment involved in the production of a particular product.

### 1.1. Process Intensification, Membranes and Membrane Reactors

**Figure 3:**
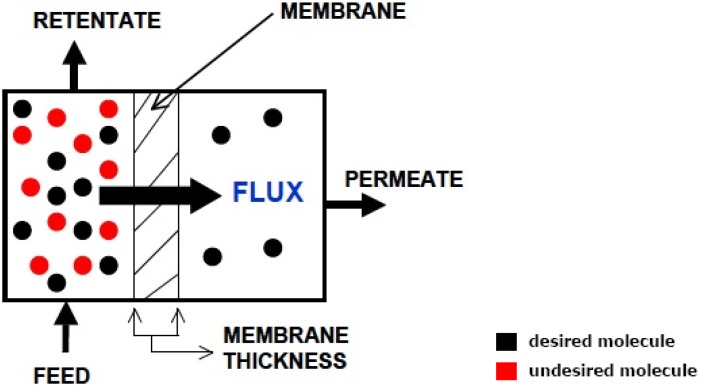
A schematic of a membrane unit for separation.

**Figure 4:**
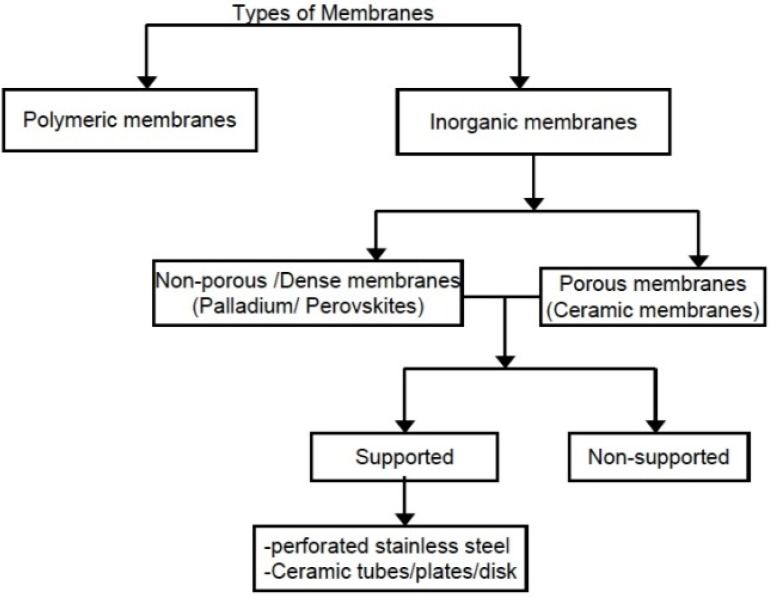
General classification of membranes.

According to the IUPAC definition, a membrane reactor (MR) is a device combining a membrane-based separation and a chemical reaction in one unit [4]. Also, MR is a device for carrying out a reaction and a membrane-based separation simultaneously in the same physical enclosure or in close proximity [5]. A membrane is defined essentially as a barrier, which separates two phases and restricts transport of various chemicals in a selective manner (see Figure 3). A membrane can be homogenous or heterogeneous, symmetric or asymmetric in structure, solid or liquid; it can either carry a positive or negative charge or it can be neutral or bipolar. General classification of membranes is depicted in Figure 4. Unlike most chemical engineering separation processes which are governed by phase equilibrium relations, membrane separation is based primarily upon the relative rates of mass transfer. Transport occurs by a solution-diffusion mechanism (for liquid separation) or adsorption-diffusion mechanism (for vapor/gas separation) and membrane selectivity is based upon the relative permeation rates of the components through the membrane. Each component (gas/vapor) transporting through the membrane has a characteristic permeation rate that is a function of the ability to dissolve and diffuse. The two relationships upon which the equations are based are Fick’s Law (diffusion) and Henry’s Law (solubility). The transport through a membrane can be affected by convection or by diffusion of individual molecules, induced by an electric field or concentration, pressure or temperature gradient. Stages involved in the transport of a molecule through membranes are:

- adsorption of the molecule onto the interface of the high-pressure side of the membrane;
- dissolution of the molecule into the membrane at the interface and diffusion of the molecule through;
- the elution of the molecule from the membrane at the interface;
- desorption of the molecule from the low pressure side of the membrane.

**Figure 5:**
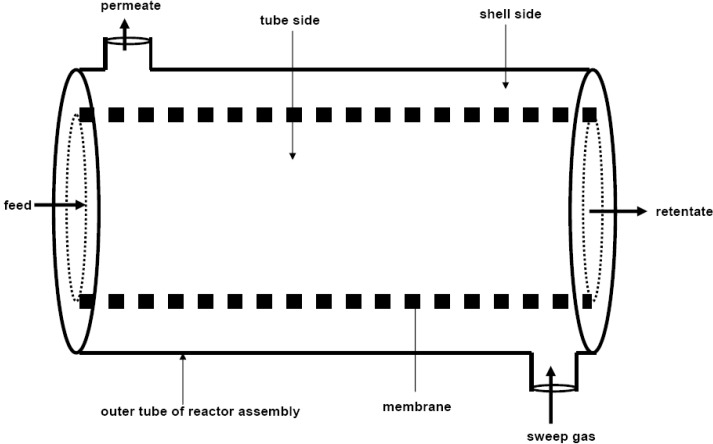
Membrane reactor concept for reaction coupling separation process intensification and its components for membrane reactor concept for reaction coupling separation.

Concept of membrane reactor for reaction coupling separation, embracing process intensification, is depicted in Figure 5. In the concept, membranes are applied for selective removal of the target product from the reaction zone.

Early works on the application of membrane-based reactive separation, in which the membranes were also catalytically active, made use of dense metal membranes. Examples of early studies on membrane-based reactive separation are presented in Table 1.

**Table 1:** Early studies on the application of membranes on reactive separation.

Membrane system	Reaction	Reference
Pd-Alloy membrane reactor	Dehydrogenation of hydrocarbons	[6]
Pd-Rh foil membrane	Dehydrogenation of cyclohexanediol to pyrocatechol	[7]
Pd-Ru-Ni Alloy membrane	Dehydrogenation of isopropanol	[8]
Pt/Al_2_O_3_-Pd membrane	Dehydrogenation of cyclohexane to benzene	[9]

Meanwhile, little progress has been made in commercializing these processes over dense metal membranes because of their limitations such as cost, fabrication techniques, durability and catalyst poisoning [10]. To overcome the limitations associated with palladium-based (Pd-based) membranes for separation coupling reaction application, usage of porous metals was proposed. A nice review on the fabrication of modified Pd-based membranes, containing porous metals such as copper, indium, ruthenium, yttrium and lead as alloys, has been published [11]. The presence of the metal alloys enhances membrane permeability and corrosion resistance of the membranes [11]. The modified Pd-membranes were applied in a series of reaction coupling separation applications such as hydrogenation of styrene to ethylbenzene, p-carboxybenzaldehyde to p-toluic acid at 254 °C and 5.4 MPa; nitrobenzene to aniline at 250 °C ( with 100% yield) and nitroethane to ethylamine at120 °C (resulting in 100% yield) [10].

Furthermore, applications of polymer composite membranes in reactive separation operations have been reported [12,13,14,15,16]. Porous polymer membranes offer a lot of advantages compared to palladium-based membranes but their applications are limited due to their poor thermal and chemical resistance, poor durability and catalytic deactivation [10,17]. Also organic membranes are characterized by decomposition or failure above 100–300 °C when used as supports for catalytic membranes [10].

**Figure 6:**
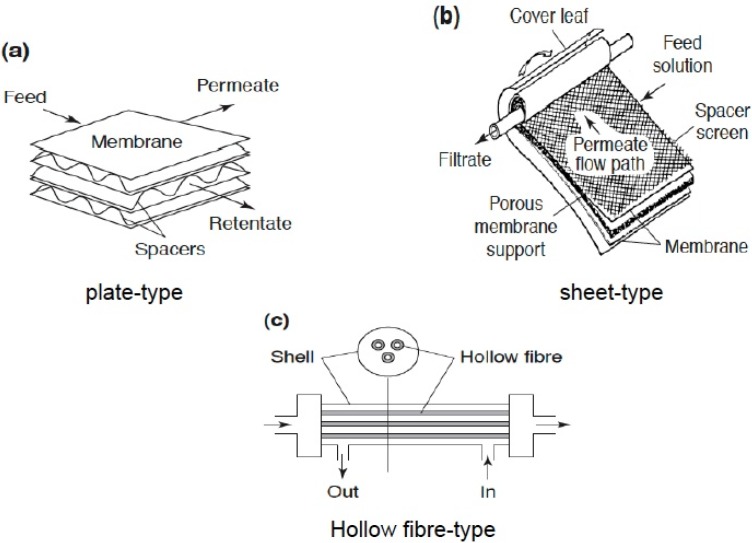
Types of membrane configurations: (**a**) plate-type; (**b**) sheet-type; (**c**) hollow fiber-type.

As a result of limitations associated with organic membranes, inorganic membranes based on zeolitic material have been developed so that the realization of the concept of a catalytic membrane reactor is increasingly possible. General comparison of organic membranes and inorganic membranes is provided in Table 2. Also, various membrane configurations depicted in Figure 6, like plate-type, sheet-type and hollow fiber, have been developed and investigated. The membrane thickness may vary from as small as 10 microns to few hundred micrometers. In some cases, the driving force in membranes is pressure difference, resulting in dramatic reduction in heating/cooling costs in comparison to crystallization or adsorption techniques. Furthermore, due to the modular nature and robustness of these membranes, they can be integrated easily in already existing chemical plants, offering the possibility of continuous operation without requiring sorbent regeneration.

**Table 2:** Comparison between organic and inorganic membranes.

Ceramic membranes	Polymeric membranes
Do not swell	Do swell
Possibility of uniform, molecular sized pores allowing for molecular sieving	Do not have uniform molecular sized pores
Chemically resistant to solvents and low pH	Not chemically stable. Denatured at low pH
Thermally stable	Not thermally stable, denatured at high temperature
High cost of production	Lower cost of production
More brittle	Less brittle

### 1.2. Zeolite, Zeolite Membranes and Zeolite Membrane Reactors

#### 1.2.1. Zeolites

**Figure 7:**
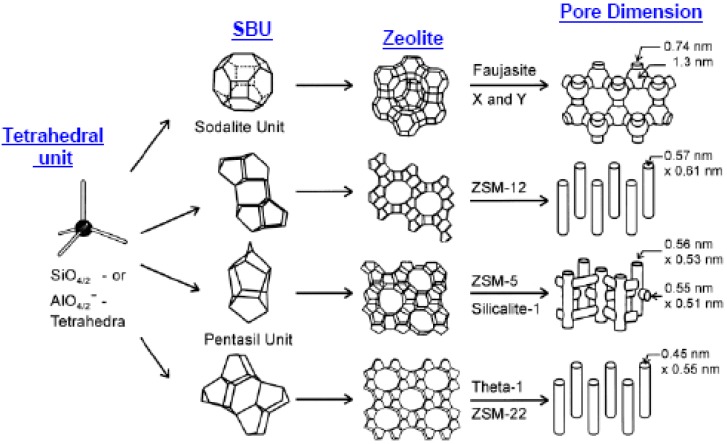
Development of zeolite structures (from aluminosilicates to secondary building blocks to zeolite structures) showing zeolite X & Y; zeolite ZSM-5 or silicalite-1; zeolite ZSM-12 and zeolite Theta-1 or ZSM-22 (adapted from Reference [24]).

**Figure 8:**
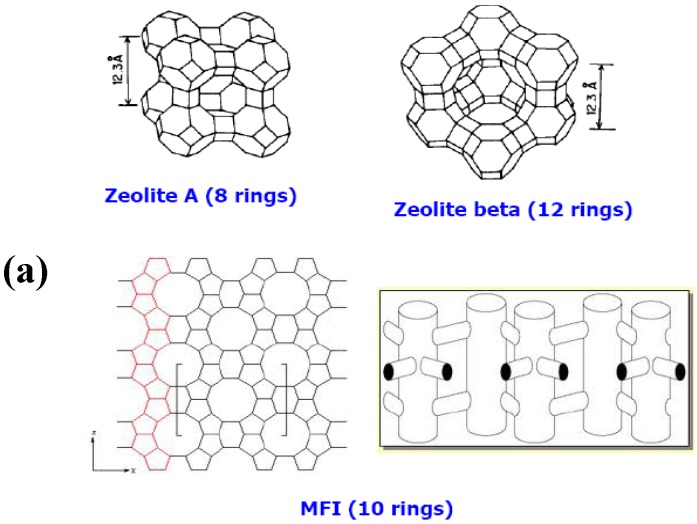

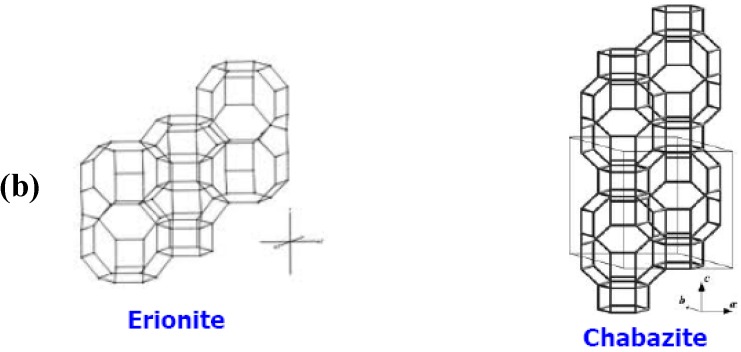
Zeolite structures showing; A: 8-ring, 10-ring and 12-ring members and; B: other types of zeolite structures.

Zeolites have been the major materials employed in the fabrication of inorganic membranes for separation/reactive-separation applications and several reviews on their synthesis and applications have been documented in the open literature [18,19,20,21]. Zeolites are tridimensional microporous crystalline aluminosilicates. The crystalline aluminosilicates consist of Si and Al tetrahedral units (TO_4/2_, where T = Si or Al) linked through bridging oxygen atoms giving rise to the so-called secondary building units (SBUs), constituted by rings and prisms of various sizes [22] (see Figure 7). These units combine to generate frameworks with a regular distribution of molecular-sized pores and cavities. The general formula of zeolites is M_x/n_[(AlO_2_)_x_ (SiO_2_)_y_].zH_2_O, with M defining the compensating cation (usually from groups I or II) with valence n. The Si/Al ratio of the zeolite structure and amount of cations control the surface properties of zeolites (e.g., hydrophobicity and acidity), and determine their adsorbent, catalytic and ion-exchange properties. Comparing zeolite with other porous materials like activated carbon, activated alumina, or silica gel, reveals that pores of zeolites are uniform in sizes determined by their crystal structures [22]. Furthermore, depending on the interconnection between the SBUs and the oxygen bridges, zeolite structures are categorized into 8-membered, 10-membered and 12-membered ring pores [23]. For instance, the pentasil chains (SBUs of ZSM-5) are interconnected by oxygen bridges to form corrugated sheets with 10-membered ring holes. Like the pentasil units, each 10-ring hole has Al or Si as vertices with an O bonded between each vertex. Each corrugated sheet is connected by oxygen bridges to form a structure with “straight 10-ring channels running parallel to the corrugations and sinusoidal 10-ring channels perpendicular to the sheets (see Figure 7 for more details). Examples of other categories of zeolites are depicted in Figure 8.

Transport of molecules within zeolite crystals is controlled by an adsorption-diffusion mechanism. In zeolites, two types of diffusivities have been identified: transport diffusivities (Fickian diffusivities) and self diffusivities [25]. Transport diffusivities (*D_T_*) are measured under *non-equilibrium* conditions in which finite gradients of loading exist (*∇q_T_* *≠* *0*), while self-diffusivities are measured under *equilibrium* conditions (*∇q_T_* *=* *0*) where finite gradients of loading do not exist and involve mass transfer of identical but labeled molecules. Through modeling studies of transport of molecules in zeolites using Monte Carlo (MC) and Molecular dynamics (MD), it was discovered that self-diffusivities agree fairly well with the values measured by microscopic methods while the diffusivities measured by macroscopic methods are often found between one and three orders of magnitude lower than the values measured by microscopic methods [26,27]. The discrepancy has been attributed to anisotropic diffusion behaviors of molecules through zeolites [28].

Using time-resolved FTIR spectroscopy, Muller *et al* have shown that diffusion of molecules in zeolite (e.g., p-Xylene diffusivity on silicalite-1 single crystals) is about three orders of magnitude higher than the value measured on polycrystalline samples [29]. With the use of a frequency response method (FR), Song *et al* reported self-diffusivities between 1 and 3 orders of magnitude higher in spherical (twinned) than in cube-shaped silicalite-1 particles at the same loading and temperature conditions for the molecules (e.g., p-Xylene) [30], indicating the influence of the morphology of zeolite on the diffusion of molecules through it.

When surface diffusion along the surface within the zeolite pores is the rate limiting step in the transport mechanism, generalized Stefan-Maxwell theory (SM) developed by *Krishna* from mixture diffusion on bulk fluids could provide an adequate basis for the description of multi-component mass transfer of adsorbed species in zeolites [26,31]. Stefan-Maxwell (SM) theory assumes that movement of a species is caused by a driving force, which is balanced by the friction experienced from the other species and the pore walls (since the size of the permeating molecules is of the same order as that of the micropores). Therefore, the general form of the SM equations applied to surface diffusion is:

(1) −ρpqiRT ∇Tμi=∑j=1j≠iC(qj NiS−qi NjS)qM,j DijS+NiSDiVS i,j=1,…,C

where *q_i_* and *N_i_^S^* are the molar loading and the surface flux of the *i^th^* species, respectively; *∇_T_μ_i_*, is the driving force; *Đ_ij_^S^* , S*M* counter exchange diffusivities; *Đ_iv_^S^*, SM surface or “jump” diffusivities. In Equation (1), SM diffusivities rather than Fickian or transport diffusivities are used because surface fluxes are related to chemical potential gradients instead of loading gradients. The first term on the right-hand side in Equation (1) reflects the friction exerted between two sorbate molecules, while the second term represents the friction between a sorbate molecule and the pore wall. These interactions were modeled by means of *SM* counter exchange diffusivities, *Đ_ij_^S^*, and SM surface or “jump” diffusivities, *Đ_iv_^S^*, [32]. If *Đ_ij_^S^* →∞ , the first term on the right-hand side of Equation (1) vanishes, implying that the surface motion of the sorbate species i does not affect the motion of sorbate species j.

Mechanistically, the SM surface diffusivity, *Đ_i_^S^*, is related to the displacement of the sorbate molecules, *λ*, and the jump frequency, *ν(q^T^)* (for strongly confined aromatic molecules is expected to depend on the number of occupied sites, *q^T^*) as follows [32]:

(2) DiS(qT)=1z λ2 νi(qT)=1z λ2 νi(0) f(qT)

The expression of function *f(q^T^)* depends on the degree of confinement of the diffusing molecules within the zeolite host and on the sorbate-sorbate interactions [33]. In the case of multicomponent diffusion, the *SM counter exchange diffusivities* can be modeled using the *Vignes* relationship [32]:

(3) DijS(qT)=[DiS(0)] θi/(θi+θj) [DjS(0)] θj/(θi+θj)

With the application of Arrhenius-type equation, temperature-dependence of the SM surface diffusivities at zero coverage, *Đ_i_^S^(0)* can be modeled according to Equation (4) [34,35]:

(4) DiS(0)=Di,T→∞S(0) exp(−EiSRT)

where Di,T→∞S(0) = *A_i_^S^* , the pre-exponential factor and *E_i_^S^* is the activation energy.

Furthermore, Krisha and co-workers expressed the surface chemical potential gradients in terms of the molar loadings gradients with the introduction of the matrix of thermodynamic factors, *Γ_ij_*, determined by the form of the mixture adsorption isotherm [32,36]:

(5) $$\frac{q_{i}}{RT}\;\nabla \mu_{i}=\sum_{j=1}^{N}\Gamma_{ij}\;\frac{q_{i,M}}{q_{j,M}}\;\nabla q_{j}$$

(6) $$\Gamma_{ij}\equiv \;\left(\frac{q_{j,M}}{q_{i,M}}\right)\;\frac{q_{i}}{P_{i}}\;\frac{\partial P_{i}}{\partial q_{j}}$$

Under weak confinement, Equations 5 and 6 are combined with the single-site Langmuir isotherm to derive the thermodynamic factors, *Γ_ij_*, resulting in Equation 7 [37,38]:

(7) J=ε ρMFI DS(0) qMτℓln[1+K PR1+K PP]

where $P_{R}$ and $P_{P}$ are the retentate and permeate pressures, respectively and *K* and *Đ*_i_^S^ are the adsorption constant and the SM surface diffusivity at zero loading:

(8) $$K=exp\left[\frac{\Delta S{}^{\circ}}{R}-\frac{\Delta H{}^{\circ}}{RT}\right]$$

(9) DiS(0)=Di,T→∞S(0) exp(−EiSRT)

Furthermore, it has been shown that molecular locations in the channels and intersections of zeolite (e.g., silicalite-1) during equilibrium adsorption in zeolite membranes is significantly affected by the molecular shape and size of the component, resulting in different adsorption behaviors [39]. Therefore, various adsorption models, *i.e.*, Langmuir isotherm, dual site Langmuir isotherm, extended Langmuir isotherm, ideal adsorbed sorption theory (IAST) and real adsorbed sorption theory (RAST) have been applied to describe the adsorption isotherms for single component, binary mixtures and multicomponent mixtures in zeolite membranes [39,40,41,42,43,44,45]. Therefore, permeation of molecules through zeolite membranes could be predicted based on the pure component Stefan-Maxwell diffusivities and adsorption parameters [46]. The quantification of the adsorption parameters is the basic information that is needed to model the transport mechanism of molecules through a zeolite membrane. The energies of interaction between the molecule and the membrane will also provide an estimate of the ease of adsorption of each component on the membrane [47,48,49,50,51,52]. Understanding this mechanism has been demonstrated for membrane separation of xylene isomers and CO_2_/N_2_ using silicalite-1 membranes [53,54,55,56]. Extensive studies of adsorption and diffusion of hydrocarbon on silicalite-1 membrane have been reported by Krishna and coworkers [44,57,58,59,60,61].

Also, factors influencing selectivity of membrane separation have been identified as: (i) differences in the adsorption characteristics of the individual species, and (ii) differences in the mobilities (diffusivities) of the components [62]. Thus, the characteristics of the zeolite membrane dominate the transport of molecules through the membranes [37].

#### 1.2.2. Zeolite Membranes

**Figure 9:**
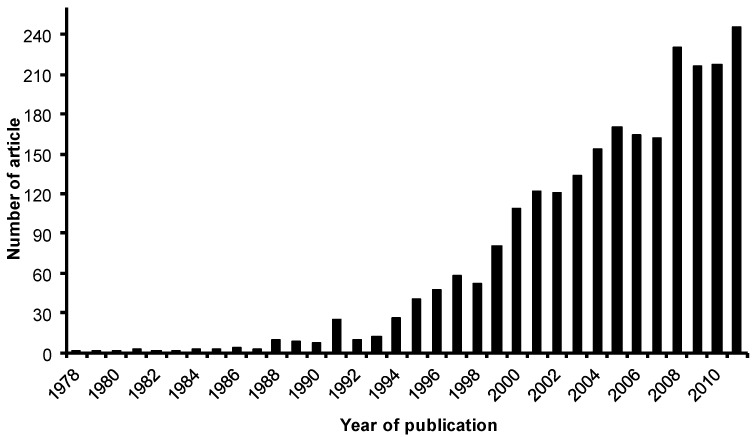
Trends of scientific research on development of zeolite membranes between 1978 and 2011 (obtained from Reference [74]).

Reports on the fabrication of zeolite membranes dates back to the late 30s [63,64,65]. In a study, Marshall and his co-workers demonstrated the application of zeolitic membrane electrodes (4–12 mm diameter, 1 mm thick) made of apophyllite and chabazite for electrical potential measurement. Since this time, more than 2534 articles have been published in scientific journals with about 185 articles per year between 2000 and 2011 (see Figure 9). The zeolite materials that have been extensively studied for membrane applications include zeolite X & Y, zeolite MFI (ZSM-5 and Silicalite-1), zeolite Na A and sodalite [53,54,55,56,66,67,68,69,70,71,72,73].

Several strategies have been proposed and used to prepare zeolite membranes [69,70,71]. Basically, all methods described in the literature involve either the use of zeolite crystals previously synthesized or the crystallization of zeolite layers. A quick overview of the most commonly studied zeolite membrane synthesis techniques is presented in Table 3.

**Table 3:** Most commonly studied zeolite membrane synthesis techniques.

Synthesis technique	Description	Reference
Liquid-phase hydrothermal (LH) synthesis technique (*in situ* hydrothermal synthesis) (LH)	One-step deposition of a layer containing the Si and Al precursor as a dry amorphous aluminosilicate gel onto a support using sol-gel technique followed by zeolitization under vapor	[75,76,77]
Vapor phase transport (VPT) technique	Two-step technique involving coating a support with amorphous gel containing Si and Al , followed by crystallization	[70,71]
Secondary seeded growth (SSG) technique	Two-step technique involving initial ex-situ seeding of a support by previously synthesized zeolite crystals followed by hydrothermal crystallization	[78,79,80,81,82]
Pore-plugging hydrothermal (PH) synthesis technique	One-stage technique involving growing zeolite crystals within pores of a support until the pores are completely blocked the zeolite materials	[53,54,55,66,67,68]

Liquid-phase hydrothermal synthesis is a simple technique but its success in yielding reproducible zeolite membranes depends on the surface properties of the support which are difficult to control. Also nucleation competes with growth process, thereby limiting nuclei density due to the mass-transfer problem. With this technique, high selective membranes are fabricated by growing a thick zeolite layer, leading to formation of cracks. With VPT, control of nucleation and growth process, which compete with each other, is possible but formation of cracks in the amorphous layer is a major set-back towards obtaining reproducible membranes. Using SSG, quality and reproducibility of zeolite membranes are enhanced due to decoupling of the nucleation and growth processes as a result of *ex-situ* seeding. However, unavailability of a dependable and perfect seeding technique to form uniform seed distribution on the surface of support is a major problem. With PH, fabricated membranes consist of separative continuous composite zeolite membranes embedded within the pores of the supports. Advantages of these membranes over conventional zeolite film membranes prepared using LH, VPT or SSG include (i) minimization of crack formation resulting from thermal expansion mismatch between the support and the zeolite crystals; (ii) easy scale-up of the preparation procedures at the commercial level as the route is less challenging than the conventional route and; (iii) easiness of membrane handling and module assembling. However, membranes from PH have low membrane fluxes but very high selectivity in comparison to membranes from the other three techniques [53,54,55,56,83,84].

Despite the extensive research on the synthesis and application of selective zeolite membranes, reproducibility remains a major problem. Although zeolite membranes have displayed very high separation performance compared to those of pure polymeric membranes, the costs of ceramic support materials for zeolite-based membranes are very high, contributing to reluctance in accepting the technology on an industrial scale [85,86,87].

Zeolite membranes are thermally stable with good chemical resistance and mechanical strength in comparison to pure polymer membranes but they are very fragile and brittle. Furthermore, the technology for fabricating commercial zeolite membranes is still in the developmental stage while the technology for commercial production of polymeric membranes is very mature with applications in a series of industrial processes. For instance, commercial application of zeolite NaA (Linde A) for solvent dehydration by pervaporation has been reported [88]. Therefore, zeolite membranes are expected to be encountered for applications on a large scale, even as catalytic membrane reactors, and thus compete with existing technologies in the near future. However, the future gas separation and reactive-separation applications of zeolite membranes depend strongly on the selectivity, permeability (flux) and stability characteristics of these membranes. In addition, the development of a high-performance and energy-efficient membrane-based process will depend on the availability of highly-selective and robust zeolite membranes, fabricated with simple and cost-effective protocols [89].

To address the issue of poor reproducibility, loss of mechanical strength in polymeric membranes and huge costs of production of zeolite membranes, mixed matrix membranes have been proposed [90]. Mixed matrix membranes (MMMs) are composite membranes containing zeolite crystals embedded within the matrix of the polymers. The presence of crystals within the polymers improves separation performance, mechanical strength and thermal stability of the polymeric membranes. Advantages of MMMs over pure polymeric membranes include:

1. Elimination of seeding technique because the zeolite crystals embedded within the matrix of polymer-zeolite composite, used as supports, serve as seeds;
2. Easy formation of uniform crystal distribution, enhancing reproducibility;
3. Possibility of obtaining membranes at low temperatures, reducing energy cost;
4. Desirable mechanical properties, economical processability of the polymers;
5. Unique structure of the dispersed inorganic phase possesses unique structure, good surface chemistry and mechanical strength.

However, the chemical structure of the inorganic fillers, type of inorganic fillers and surface chemistry are mitigating factors to obtaining high quality MMMs [91].

#### 1.2.3. Zeolite Membrane Reactors

**Figure 10:**
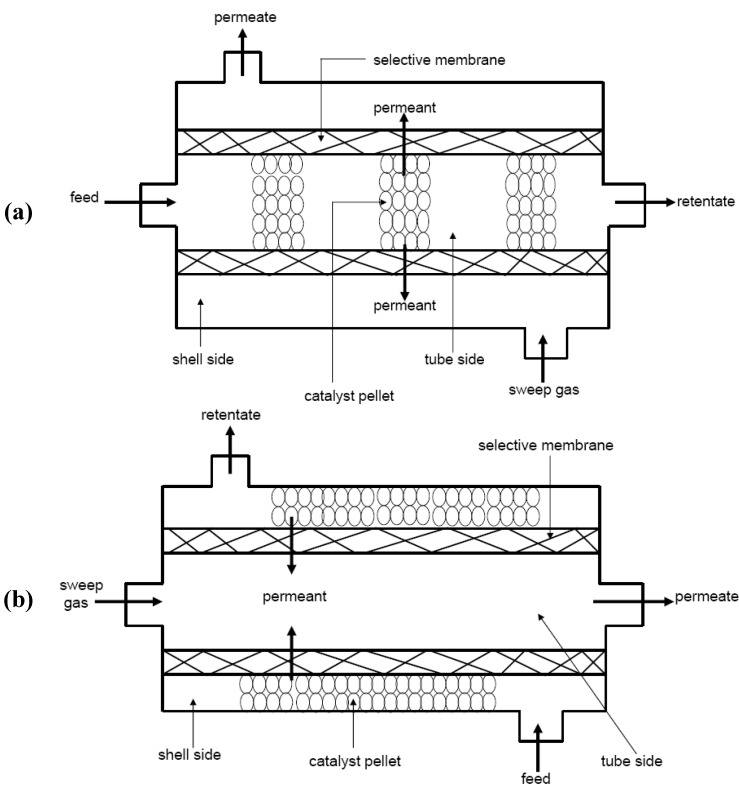
Schematic of inert zeolite catalytic membrane reactor (IZCMR) with catalyst packed (**a**) inside the tube of zeolite membrane; and (**b**) outside the tube of zeolite membrane.

Zeolite Catalytic Membrane Reactors (ZCMRs), where zeolite membrane separation is coupled with catalytic reaction in the same unit, are attractive applications because they have been demonstrated to be potentially compact, less capital intensive and have lower operating costs than more conventional processes. Two main types of zeolite membrane reactors have been identified based on the catalytic property of zeolite membranes, namely: inert zeolite catalytic membrane reactor (IZCMR) and the zeolite catalytic membrane reactor (ZCMR). In this kind of membrane reactor, the membrane is not catalytically active and does not participate in the reaction but it simply acts as a selective separation unit for the desired products while acting as a barrier to the reactants and undesired products. The IZCMR allows catalyst pellets to flow with the reactants on the feed side (usually the inside of the membrane). This type of configuration is also referred to as an Inert Membrane Reactor with Catalyst on the Feed side (IMRCF). Schematic of IZCMR is depicted in Figure 10. On the other hand, a zeolite catalytic membrane reactor (ZCMR) has a zeolite membrane that has either been coated with or is made of a material that contains catalyst. For ZCMRs, the membrane itself participates in the reaction. Some of the reaction products (those that are small enough) pass through the membrane and exit the reactor on the permeate side [92].

**Figure 11:**
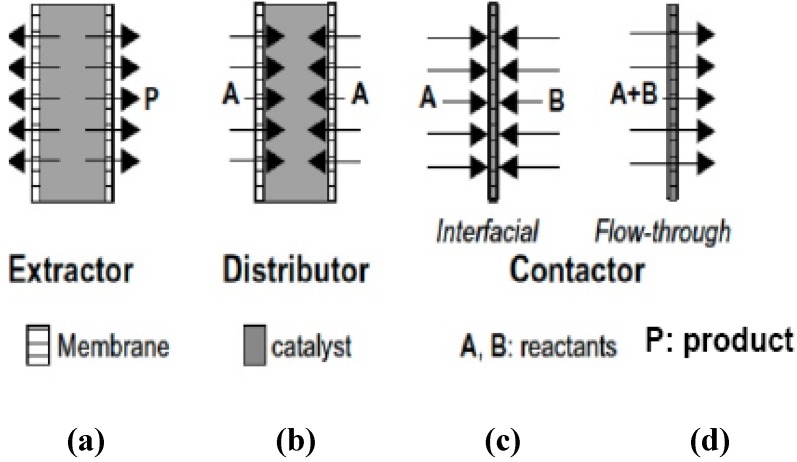
Classification of ZCMRs: (**a**) extractor-type; (**b**) distributor-type; (**c)** flow-through contactor-type; and (**d**) interfacial contactor-type.

On the basis of the way zeolite membrane and the catalyst are combined, ZCMRs have been broadly classified as: (i) extractor-type zeolite catalytic membrane reactors (e-IZCMRs/e-ZCMRs); (ii) distributor-type zeolite catalytic membrane reactors (d-ZCMRs); and (iii) contactor-type zeolite catalytic membrane reactors (c-ZCMR which also include flow-through or interfacial), these latter being operated in either flow-through or interfacial configurations [93]. Schematically, Figure 11 compares the three ZCMR configurations. In all cases, the membrane can show inherent catalytic character or only act as a separation/contactor unit between the phases and the catalyst. More specific details about these configurations and application domains can be found in the literature [94,95,96].

**Figure 12:**
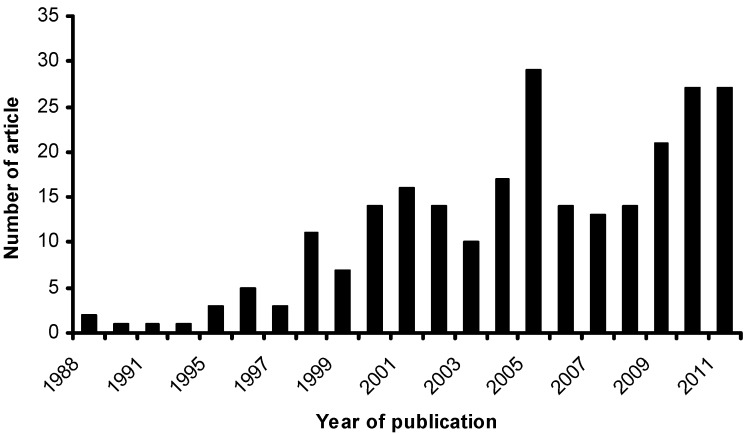
Trends of scientific research on development and applications of ZCMRs between 1988–2011 (obtained from Reference [74]).

Among the types of ZCMRs, extractor-type ZCMRs (e-IZCMR/e-ZCMR) are by far the most widespread application of ZCMRs. Classical applications of this configuration range from dehydrogenation, isomerization and esterification/etherification reactions to hormone synthesis and wastewater biological treatment. In this configuration, selective removal of one/more products from the reaction zone enhances the conversion of the reaction by shifting the equilibrium position or by promoting the catalytic activity. However, to overcome the equilibrium restriction, the reaction must be sufficiently fast compared with the mass transport through the membrane (kinetic compatibility). A special benefit can be that the removal of one of the products provides an integrated product purification thus decreasing the number of process units. Also activity improvements can be found by selectively removing reaction rate inhibitors. As a result of the need for more energy-efficient technologies in the chemical industry, research efforts on the development of ZCMRs have been intensified. Between 1988 and 2011, about 250 articles were published in open scientific journals (see Figure 12 for the progression). Analysis of ZCMRs is based on the fundamental principles usually employed for conventional reactors (e.g., plug flow reactors), except transport law accounting for the transport of molecules across the membrane is included in the mathematical expression. Basically, analysis of ZCMRs involves:

1. Mole balance in the catalytic bed : Material in – Material out + Generation = Accumulation.
2. Rate law that accounts for disappearance of reactant: $R=kC^{n}$. Where R, the reaction rate; k, reaction rate constant; C, reactant concentration and n, the reaction order.
3. Transport law accounting for the transport or flux of product through the membrane: $J=k_{m}\Delta C$. Where J, is the flux of the product through the membrane; $k_{m}$, the mass transfer coefficient; and ΔC, the concentration gradient across the membrane. It is noteworthy to mention that the transport law takes into account the adsorption-diffusion mechanism that governs the transport of molecule through zeolite membranes.

Extensive information on the analysis and modeling of ZCMRs can be obtained from the literature [97,98,99,100,101]. In spite of research efforts in ZCMRs, substantial development and understanding have been hampered by difficulty associated with fabricating and reproducing highly-selective zeolite membranes for ZCMRs applications. However with the new research line on the fabrication of mixed matrix membranes with zeolite crystals as fillers, great milestones in the development and application of ZCMRs are expected in the near future.

## 2. Potential Applications of Zeolite Membrane Reactors in Reactive-Separation

### 2.1. Synthesis of Chemicals in the Chemical and Petrochemical Industry

The development of inorganic membrane materials (Zeolites, ceramics, and metals) has broadened the application potential of ZCMRs towards the (petro) chemical industry [96]. In recent times, ZCMRs have shown their potential in solving problems of synthesis and separation in the chemical and petrochemical industry on a very small scale. Applications of ZCMRs in reactions like oxyfunctionalization of n-hexane to hexanols and hexanones [102]; catalytic hydrogenation of ethylbenzene (EB) to styrene [103]; metathesis reactions involving conversion of propene to ethene and 2-butene [104], isobutane dehydrogenation reaction in a MFI zeolite membrane reactor [105]; and isobutane dehydrogenation in a DD3R zeolite membrane reactor [106]; to mention but a few. Kong *et al*. reported an 11% increase in EB conversion in an e-IZCMR, equipped with an iron oxide catalyst, when compared with the performance of an equivalent FBR operated at similar conditions as the e-IZCMR [103]. In addition, van de Graaf *et al*. reported a 13% increase in propene conversion in the e-IZCMR, when compared with performance of an equivalent FBR operated under similar conditions [104].

In the area of reduction of CO_2_ (one of the emissions from the chemical and petrochemical industry), catalytic hydrogenation of CO_2_ into methanol has been proposed. Experimental study of the hydrogenation of CO_2_ into methanol in a ZCMR has been demonstrated [107,108]. In the study of Gallucci *et al*., an e-IZCMR equipped with zeolite NaA (LTA), prepared by *in situ* crystallization, was applied to convert CO_2_ into methanol. The membrane was supported on an α-alumina support and packed with 8 g of Cu/ZnO/Al_2_O_3_ catalyst. For comparison, the authors performed the same reaction in an equivalent conventional fixed bed reactor (FBR) operated under similar conditions as e-IZCMR. The results revealed that e-IZCMR performed better than the FBR with about 270% increase in methanol yield at a H_2_/CO_2_ ratio of three and operating pressure of 20 bar. The CO_2_ conversion and selectivity for e-IZCMR were 11.6% and 75%, respectively. For FBR, CO_2_ conversion and selectivity were 5% and 48%, respectively, indicating a 6.6% decrease in CO_2_ conversion and a 27% decrease in selectivity when compared to performance of e-IZCMR. These promising results confirm the potential of zeolite catalytic membrane reactors in reducing green-house gas.

Synthesis of para-xylene (PX), one of the petrochemical products that is almost exclusively used as raw material in the production of terephthalic acid (TPA) and dimethyl terephthalate (DMT) has been demonstrated in e-IZCMRs/e-ZCMR [83,84,99,109,110,111,112,113,114]. PX is always reacted with ethyleneglycol to form polyethylene terephthalate (PET), the raw material for polyester resin. Polyester resin is used to manufacture polyester fibers, films and fabricated items (e.g., beverage bottles). Global PX demands are expected to rise at an average rate of 7% per year in the period 2008–2013, driven mainly by TPA and PET demand increase in China, other Asian countries and in the Middle East [115]. Production of PX via isomerization is chemical-equilibrium limited [95], making conversion above equilibrium during the xylene isomerization process in conventional catalytic reactors (FBR) impossible. Therefore, existing industrial technology could only produce equilibrium or near equilibrium xylene mixtures. Recycling the xylene streams back into the process lines might ensure higher PX productivity, but at the expense of higher operational costs due to higher energy consumption.

One-stage production of ultra-pure PX from MX isomerization over a Pt-HZM-5 catalyst between 200 °C and 300 °C, using an e-IZCMR equipped with a nanocomposite zeolite MFI membrane as separating unit for MX isomerization, has been reported [83]. Also at an operating temperature of 200 °C, the authors reported 56% of MX conversion in e-IZCMR and 18% increase in PX yield in e-IZCMR, when compared with an equivalent FBR operated at similar conditions as the e-IZCMR. Recently, Gu and his co-workers reported 92% para-selectivity with about 6.5% increase in MX conversion over an equivalent FBR operated under similar conditions as their e-ZCMR [114]. Difficulty mitigating a progress with this application is inherent in the non-availability of a robust and scalable synthesis technique for producing reproducible and highly selective zeolite membranes.

Zeolite membranes prepared via pore-plugging hydrothermal (PH) synthesis techniques have been shown to be highly selective but with low membrane fluxes [54,56]. Also, reports have shown that zeolite membranes synthesized through PH are reproducible but the reproducibility is hampered by the quality of the ceramic supports [54]. In general, successful fabrication of reproducible highly selective zeolite membranes, having reasonable membrane fluxes for industrial applications, depends on:

1. Presence of cheap and high quality membrane supports. In some cases, cheap supports are modified before synthesis or membrane defects healed for enhanced membrane selectivities.
2. Optimized membrane synthesis conditions and membrane configuration that could result in very reasonable membrane fluxes. In this regard, the use of a hollow fiber membrane configuration is a promising option [56].

### 2.2. Potential Applications in the Fuel Cell System

Recent environmental concerns on t existing technologies employed for non-renewable energy generation and consumption stimulated the search for benign technologies. One of the promising options is the portable and stationary fuel cell system. Pure hydrogen is required for efficient performance of a fuel cell. Studies have shown that the presence of CO at a concentration of 10 ppm in the H_2_ feed for fuel application causes deactivation of the Pt-impregnated electrode [116], reducing the activity and efficiency of the fuel cell.

In view of this problem, production of high purity H_2_ from CO-H_2 _mixtures for fuel cell applications using zeolite catalytic membrane reactors (e.g., d-ZCMR) has been studied [117,118]. For example, selective oxidation of CO in hydrogen-rich mixtures was studied by Hasegawa *et al*. in a zeolite catalytic membrane reactor [117]. The study was aimed at pre-treating the H_2_ feed for fuel cell application.. Hasegawa *et al*. successfully demonstrated the potential application of ZCMR, composed of a Pt-loaded zeolite Y membrane made by ion-exchanging a zeolite Y membrane with an aqueous solution of [Pt(NH_3_)_4_]Cl_2_, which can reduce the concentration of CO in the H_2_ rich mixtures to 8 ppm. In the same vein, Bernardo *et al*., reported CO reduction from 10,000 ppm to 10–50 ppm using d-ZCMR operated at 200–220 °C and 6 bars [118].

In addition, the application of ZCMRs to produce high purity hydrogen through a dry reforming reaction of methane over a Rh/La_2_O_3_ catalyst has been demonstrated [119]. The authors used a ZCMR equipped with a Pd/LTA composite catalytic membrane and a Pd-Ag/LTA composite catalytic membrane supported on stainless steel for the conversion. Further progress in the application of zeolite catalytic membrane reactors to produce pure H_2_ for fuel cell application has been hampered by the inability to ensure even distribution of catalyst particles in the membrane layer. Generally, zeolite catalytic membrane reactors are about 10 times more active than when the catalyst pellets are used in the fixed-bed reactors provided that the membrane thickness and porous texture, as well as the quantity and location of the catalysts in the membrane are evenly distributed and adapted to the reaction kinetics [120,121]. Therefore, extensive experimental studies are required to develop a scalable technique to fabricate reproducible zeolite catalytic membrane reactors with adaptable reaction kinetics and even distribution of catalyst particles within the membrane layer.

### 2.3. Application in Selective Removal of Water from Industrial Processes

Almost all industrial processes (chemical, food, pharmaceutical, or otherwise) include water as a key component in one way or another. In some cases, the presence of water in these processes constitutes operational problems like unwanted side reactions, equilibrium limitations, catalyst inhibition or deactivation. Classical examples of the processes where the presence of water constitutes an operational problem include are presented in Table 4.

**Table 4:** Examples of industrial processes with water as intermediate or byproduct.

Process	Reaction	Reference
Production of N-Methylpyrrolidone (NMP) from γ-butyrolactone	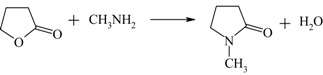	[122,123]
Tetrahydrofuran from 1,4-butanediol	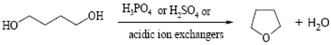	[124,125,126]
Conversion of methanol to a mixture of hydrocarbons in the Mobil process	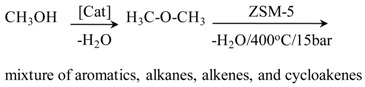	[127]
Dioctylphthalate, (DOP), from phthalic anhydride and 2-ethylhexanol	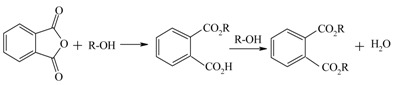	[128]
Glyoxal from ethyleneglycol	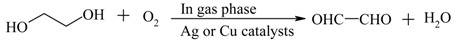	[129,130,131,132,133]
1,4-Dioxane from diglycol	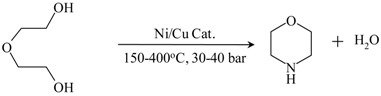	[134,135]
Morpholine from diethanolamine	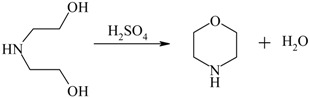	[136,137,138]
Ethylene diamine from monoethanol amine	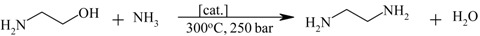	[139,140,141,142]
Esters of ethylene glycol monoalkyl ethers	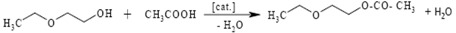	[143]
2-Vinyl picoline from 2-picoline	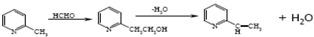	[144]
2- and 4-Picoline from acetaldehyde and ammonia	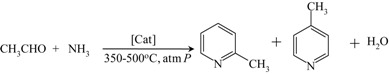	[145]
Anthraquinone from anthracene	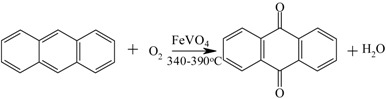	[146,147]
Benzoic acid from toluene	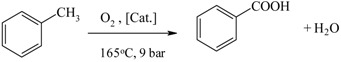	[148,149]
Butene oxidation to maleic anhydride	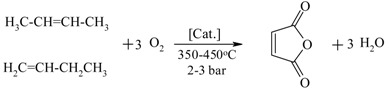	[150,151,152]
Sorbitans (monolaurate, monopalmitate, monostearate, etc.) from D- sorbitol and fatty acids	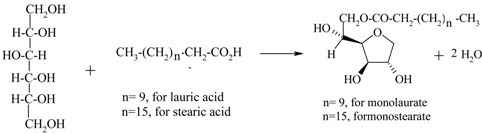	[153,154,155]

Therefore, integrated removal and purification of water plays a significant role in the operation of these processes. For thermodynamically-equilibrium limited reaction (e.g., esterification), a traditional technique to overcome equilibrium limitation is by using an excess amount of the alcohol or by separating the water (the by-product) from the reaction through reactive distillation or reactive stripping [156]. However, the use of excess alcohol may increase the operation costs on the downstream reagent recovery and result in unwanted ether formation [136], while reactive distillation is energy intensive and only useful when the products and reactants do not have close boiling points. In this respect, the use of membrane reactors is attractive as the process efficiency is not limited by the thermodynamic equilibrium conversion while the process costs can be reduced due to the smaller amounts of reactants required and the higher conversions obtained [157].

Recently, Kapteijn and his co-workers reported on the fabrication of hydroxy sodalite (H-SOD) membranes and their applications for selective removal of water from industrial processes such as dewatering of alcohols, dehydrating organic acids and desalination of sea water to produce ultra pure water with water flux (between 30 °C to 200 °C) and a water-to-ethanol separation factor of 0.2–2.3 kg.m^−2^.h^−1^ and > 10^6^, respectively [72,158,159]. The authors demonstrated the application of these membranes as e-IZCMRs for esterification-coupling separation [73]. This kind of membrane system can be applied in the Fischer-Tropsch (F-T) process to selectively remove water, a by-product that causes deactivation of the catalyst in the system.

**Figure 13:**
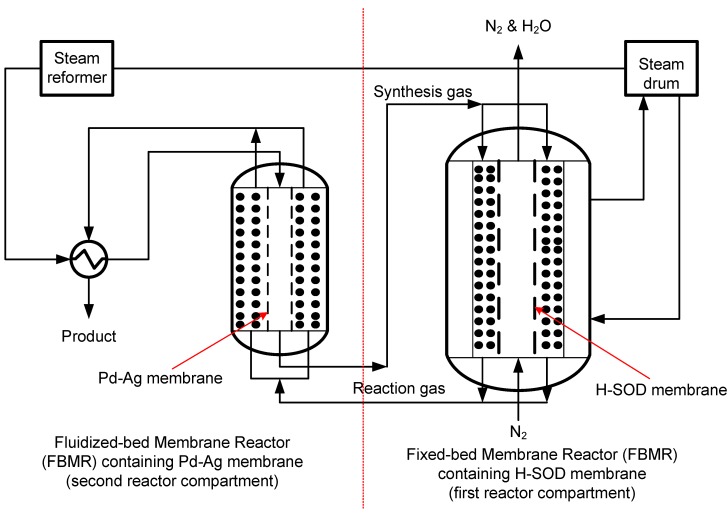
Schematic of a novel reactor configuration for F-T synthesis (adapted from Reference [162]).

The Fischer-Tropsch (F-T) process is an important chemical process for the production of liquid fuels and olefins. In recent years, a dramatic increase in the price of crude oil and increasing demand of olefins, diesel, and waxes have led to high interest in further development of this process. When iron-based catalysts are used in F-T, part of the amount of CO required for F-T is consumed by the WGS reaction, thereby influencing product distribution [160]. The Water-Gas-Shift reaction occurs in the presence of water, a by-product associated with F-T. Also, the presence of water in the reactor deactivates the catalysts. Prevention of catalyst deactivation and avoidance of WGS require *in situ* removal of water from the reaction zone. In this respect, ZCMRs equipped with hydrophilic membranes like LTA and H-SOD can be used to suppress catalyst deactivation and WGS by *in situ* removal of water. Recently, Kapteijn and his co-workers demonstrated the potential application of an e-IZCMR equipped with highly-selective H-SOD membranes in the Fischer-Tropsch (F-T) synthesis to remove water [161]. Furthermore, a novel reactor configuration referred to as fixed-bed membrane reactor followed by fluidized-bed membrane reactor (FMFMDR) has been proposed [162]. The configuration consists of an IZCMR (equipped with H-SOD membrane) and a hydrogen perm-selective membrane reactor (equipped with Pd-based membrane) (see Figure 13 for schematic). Through modeling and simulation studies, the authors reported that FMFMDR gives higher CO conversion, higher H_2_ conversion, reduced undesired products and higher gasoline yield, when compared to a conventional fixed bed reactor. However, membranes with reasonable water fluxes that could encourage industrial applications, in particular for selective removal of water from a mixture of H_2_, CO, CO_2_ and hydrocarbons under F-T conditions, are still not available [163].

Recent experience acquired through preliminary studies within our group revealed that sodalite mixed matrix membranes (SOD-MMMs) could display higher water fluxes than ceramic-sodalite membranes, but poor thermal stability of SOD-MMMs will make them impractical under F-T conditions. Therefore more experimental studies in the area of synthesis protocol and membrane configuration are essential to (i) increase water fluxes in sodalite-ceramic membranes by reducing the thickness of membrane layers or using symmetrical hollow fibers as membrane supports. Research has shown that zeolite membranes prepared on hollow fibers could enhance membrane flux by ~30% when compared to other configurations like tubular support [56]. On the other hand, membrane layers consisting of silica sodalite crystals could be prepared on the supports. Unlike hydroxy sodalite, silica sodalite crystals synthesized through topotactic transformation, possess accessible ultra-small micropores [164]. During the synthesis of silica sodalite via topotactic transformation, water molecules occluded inside sodalite cages are removed without collapsing the framework, thereby enhancing the passage of water molecules or other molecules (e.g., H_2_ and NH_3_ gases) through the cages. However, integrating the protocol of topotactic conversion into existing membrane synthesis techniques for fabricating sodalite-ceramic membranes could be a difficult task. In addition, silica sodalite crystals can be used as fillers in SOD-MMMs to enhance membrane fluxes of the membranes.

### 2.4. Application in Water Treatment and Purification Industry

Endocrine disrupting chemicals (EDCs) are chemical compounds commonly found in domestic wastewater which survive conventional wastewater treatment processes to end-up in finished, potable water [165]. Ozone and membrane processes have been proven promising options to removing EDCs [166]. Application of ozone treatment alone does not result in effective EDC treatment due to a slow mineralization rate [167]. Furthermore, several research efforts have combined an ozone treatment technique with ultraviolet radiation (UV) (UV/O_3_) and hydrogen peroxide (O_3_/H_2_O_2_) but these techniques are complex and very expensive to maintain [168,169]. Therefore, an advanced but economical technique is required to achieve total degradation/removal of EDCs.

Advances in membrane technology together with benefits of process intensification (PI) have paved the way for the application of zeolite membranes/membrane reactors in water purification and wastewater treatment and thus revolutionized the industry. Potential applications of zeolite membranes in water purification (e.g., desalination of sea water to produce ultra pure water [72,73,157,158] and as zeolite catalytic membrane reactors in wastewater treatment) have been show-cased [170,171,172].

**Figure 14:**
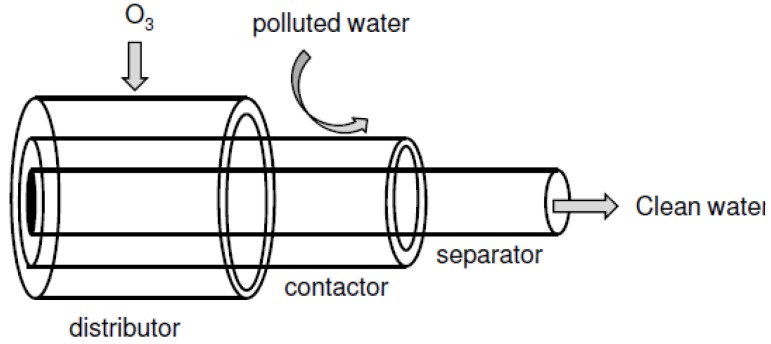
Schematic of a proposed reactor configuration for ozone zeolite membrane reactor for water treatment (adapted from Reference [171]).

Recently, Chan *et al*. proposed a membrane configuration for an ozone membrane reactor. The membrane reactor consists of three (3) tubular membrane compartments, namely: (i) distributor; (ii) contactor; and (iii) separator. The compartments are arranged concentrically with the separator situated in the innermost part of the reactor. The contactor and separator are composed of silicalite-1 membranes prepared on an α-alumina support by a seeded secondary growth technique. The schematic of the ozone zeolite membrane reactor is depicted in Figure 14. The ozone membrane reactor was used in the treatment of EDCs and the performance compared with that of a traditional semi-batch reactor. The authors reported a 30% increase in total organic carbon (TOC) removal, when compared with the performance of the traditional semi-batch reactor.

### 2.5. Application in the Bio-Refinery Industry

**Figure 15:**
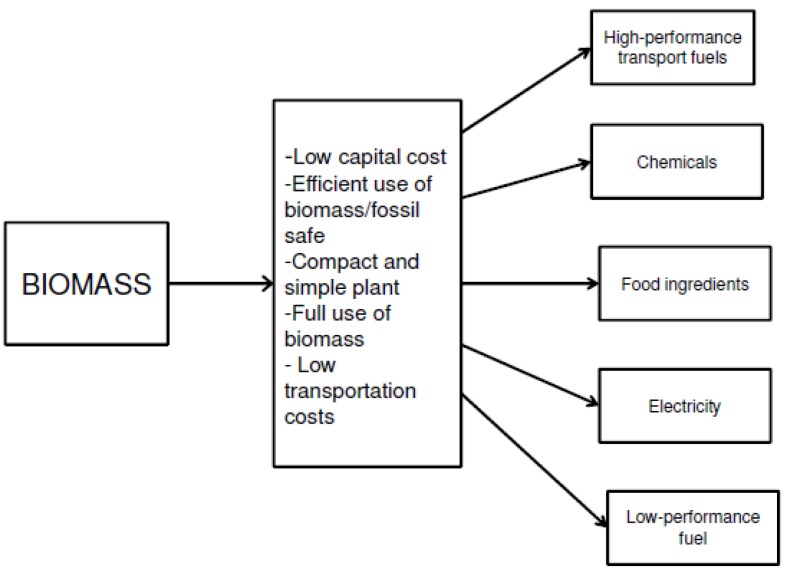
Scheme for biomass conversion and its associated products (adapted from Reference [175]).

A bio-refinery is an integrated processing facility that converts biomass to fuels, power, and value-added chemicals [173]. It is also a “catch and release” method for using carbon that is beneficial to both the environment and the economy [174]. A scheme for usage of biomass and its associated products is depicted in Figure 15.

In bio-refinery and biotechnological applications, membrane processes are coupled with industrially important biological reactions, for example, in the fermentation of amino acids, antibiotics, and other fine chemicals. Here the advantage is the continuous elimination of metabolites allowing for high reactor productivity. In other applications bacteria, enzymes, or animal cells are immobilized onto the membrane and used to produce high value chemicals and pharmaceuticals. Also, reaction coupling separation processes are currently finding use in the biological treatment of contaminated air and water streams [176].

Biodiesel production is a “hot research area” today because biodiesel could replace conventional diesel due to numerous benefits such as environmental friendliness, renewability, and biodegradability. Efforts are being made by these researchers to look into the best technology and type of reactor that will give outstanding yields of biodiesel. Biodiesel fuel is produced via different techniques such as: direct blends, microemulsion, pyrolysis, and transesterification. Transesterification is the most adopted of all these methods, and is usually catalyzed by either homogeneous or heterogeneous catalysts.

Biodiesel has been conventionally produced using reactors such as batch reactors, CSTR and plug flow reactors [177]. However, commercial production of biodiesel fuel via batch reactors is being discouraged due to the tedious mode of operation and high cost of production [177]. For example, the main drawback of the continuous stirred tank reactors or tubular reactors is that the temperature of the reaction is restricted to the boiling point of the alcohol; 65 ^o^C for methanol, if the reactor is operated at atmospheric pressure. Also for an industrial size reactor, significant mass transfer resistance is expected even when higher shear mixing is employed [178].

It has been shown that an inert zeolite NaA membrane packed with montmorillonite K10 catalyst and applied as IZCMR can be used to synthesize solketal [179]. According to Roldán *et al*. [179], the methyl esters do not suffer any side reaction during the synthesis and zeolite NaA selectively removed water that could deactivate the catalyst from the reaction zone.

A series of research has shown that the esterification of acetic acid with ethanol can be successfully carried out in continuous IZCMR equipped with mordenite or zeolite A membranes [180]. Amberlyst^TM^ 15 catalyst was packed inside the lumen of the membranes and the results showed an increase in conversion resulting from forward-shifting of the equilibrium. Furthermore, mordenite-based membranes showed a great resistance to the acidic reaction medium and operational stability with conversions ~90% maintained for five days. Recently, zeolite Na-X synthesized from coal fly ash was used to produce biodiesel in up to 85% yield [181]. This yield was found to be comparatively higher than the tested commercial zeolite by these authors. The synthesis of zeolite from fly ash source, in addition to solving the disposal problems of fly ash in countries such as South Africa and India, holds promise in the development of ZCMRs from such valued products to advance research in heterogeneous catalysis for biodiesel production. Concerted effort should be directed towards the scale-up synthesis of valued zeolites, relevant for this application, from such waste materials.

## 3. Conclusions and Future Outlook

Due to the demonstrated potential applications of a membrane-based reactive separation process coupled with the higher energy-efficiency and environmentally-friendly operations when compared to conventional/ traditional processes such as reactive distillation and fixed-bed reactor, the development of an industrial process based on the application of membrane-based reactive separation for chemical production can be foreseen in the near future. In bio-refinery, for example, in the cyclization and methylation of γ-aminobutyric acid (GABA) to N-methlpyrrolidone (NMP) [182], zeolite catalytic membrane reactors (ZCMR) equipped with LTA or H-SOD membranes can be used for selective removal of water from the reaction zone.

**Figure 16:**
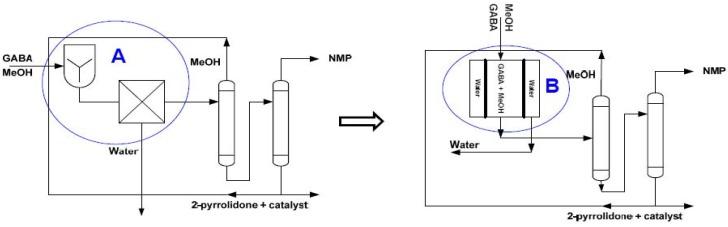
Process for cyclization and methylation of γ-aminobutyric acid by Lammens *et al*. (left-hand side) (adapted from Reference [182]). Proposed scheme with encircled region A in the process of Lammens *et al*. replaced by ZCMR, encircled region B in the proposed scheme.

A scheme of this proposition is depicted in Figure 16. In this scheme, the two process units in the encircled region A can be combined into a process unit using IZCMR equipped with a LTA/H-SOD membrane as the separation unit.

Despite vast research efforts in the area of zeolite membranes and zeolite catalytic membrane reactors, commercialization of the technology is still a mirage. To fast-track and actualize commercialization of this technology, research efforts should be channeled towards:

1. Zeolite membrane synthesis and optimization. Reaction coupling separation using ZCMRs requires highly-selective and defect- free zeolite membrane prepared through a robust and scalable reproducible technique. Also the zeolite membranes should display reasonable membrane flux for commercialization. However to enhance separation and catalytic performance of ZCMRs, factors like geometry and operational conditions, have to be optimized. Recently, further advances in catalytic membrane reactors and reactions have resulted in development of selective zeolite membranes with hollow fiber configurations. These membrane configurations offer great advantages as the hollow structure can be packed with catalysts for catalytic processes with separation occurring simultaneously [183]. In comparison with conventional membranes, hollow fibers have a larger surface area-to-volume ratios >3000 m^2^/m^3 ^and a thinner membrane wall, resulting in about 30% increase in membrane flux , when compared with membrane tubes fabricated using the same synthesis technique [56]. In addition, several hollow fibers can be made into fiber bundles, thereby reducing both the size and cost of the permeating modules for selective water removal from industrial processes. Therefore, improvement is required in this line to make the incorporation of the catalytic centre into the membranes possible without unnecessarily increasing the thickness of the inorganic supports, promoting permeability without forming pinholes or cracks. At the same time, limitations in terms of uniform temperature control and heat transfer may be overcome.
2. Zeolite membrane reactor configuration and reactor analysis. To avoid formation of undesired products in IZCMRs and thus enhance the yield, conversion and overall reactor performance, the reaction rate to membrane flux ratio should approach one. For example in PX isomerization, if the reaction rate > membrane flux (in the case of packed-bed ZCMRs), PX is isomerized to undesirable products like o-xylene and m-xylene. On the other hand, if the membrane flux > reaction rate, isomerization of m-xylene to p-xylene is affected. Therefore a suitable reactor configuration is essential to strike a balance between membrane performance and catalyst performance. In general terms, ZCMRs could be about 10 times more active than IZCMRs provided that the membrane thickness and porous texture, as well as the quantity and location of the catalyst in the membranes are adapted to the reaction kinetics [120,121]. Research efforts are still limited in the development and application of ZCMRs due to challenges in ensuring homogenous distribution of catalytic particles/layer on the membranes.
3. Zeolite membrane and zeolite membrane stability. Although zeolite membranes and zeolite membrane reactors can be employed at high temperature and chemically harsh conditions, their long-term thermal stability and operational stability under real operating conditions require significant improvement to attract industrial acceptance. Most of the fabricated zeolite membranes are thermally stable up to 400–500 ^o^C. However, some industrial applications occur at higher temperatures, requiring high thermally stable membranes. Efforts are required to produce such membranes to extend future applications of ZCMRs.
4. Techno-economical feasibility and scale-up studies. Techno-economical feasibility studies of ZCMRs are essential. The studies will lucidly elucidate the comparative advantages of the technology over existing conventional technologies. Virtually, all research efforts reported on the development and application of ZCMRs are still limited to laboratory scale studies. In view of this, scale-up studies of the technology are necessary to evaluate the competitiveness of the technology with existing processes to fast-track commercialization of the technology.

Considerable progress has been made on the synthesis of zeolite membranes and the application of the membranes as zeolite catalytic membrane reactors in several model industrial applications such as the production of fine and bulk chemicals, Fischer-Tropsch synthesis, purification of H_2_ gas for fuel cell application, ozone wastewater treatment, biofuel production and bio-refinery etc. However, further progress depends on the development of more stable, high flux and affordable zeolite membranes as well as careful process design and reactor analysis. Recent advances in synthesis of nanomaterials and application of such materials in membrane technology have led to the application of metal organic frameworks (MOFs) in membrane technology. In comparison to zeolites, MOFs cover a much wider pore size range. Pore size range of MOFs bridges micro- and mseoporous materials, making them nanometrials with unprecedented topological richness [85]. MOFs possess a combination of organic and inorganic building blocks that give them enormous flexibility in pore size, shape and structure when compared to zeolites. Porosity in MOFs is > 90% higher than in zeolites and research has shown that some of them are thermally stable, even in the presence of steam, up to 400 °C [184,185]. These materials, when studied in membrane reactors, might fast-track the development of the technology for the industrial scale.

## Supplementary Files

Supplementary File 1

Supplementary File 2
