# *Correction*: Daramola, M.O.; Aransiola, E.F.; Ojumu, T.V. Potential Applications of Zeolite Membranes in Reaction Coupling Separation Processes. *Materials* 2012, *5*, 2101-2136

**DOI:** 10.3390/ma6041432

**Published:** 2013-04-02

**Authors:** Michael O. Daramola, Elizabeth F. Aransiola, Tunde V. Ojumu

**Affiliations:** 1 Biochemical and Reactions Engineering Group, Department of Chemical Engineering, Obafemi Awolowo University, Ile-Ife 220005, Osun State, Nigeria; E-Mail: aransiola4@yahoo.com; 2 Catalysis Engineering Section, Delft University of Technology, Julianalaan 136, 2628 BL, Delft, The Netherlands; 3 Department of Chemical Engineering, Cape Peninsula University of Technology, Cape Town 8000,South Africa; E-Mail: ojumut@cput.ac.za

Due to an oversight by the authors, Table 4 in the aforementioned review article [1] should have been referred to as obtained from reference [2]: 

Khajavi, S. Separation of Process Water Using Hydroxy Sodalite Membranes. PhD Thesis, Delft University of Technology, Delft, The Netherlands, 2010.

Furthermore, an additional affiliation for the first author of this review is added as follows:

Catalysis Engineering Section, Delft University of Technology, Julianalaan 136, 2628 BL, Delft, The Netherlands

The authors apologize for any inconvenience this may have caused.
