# Supplementary material for: Potential Applications of Zeolite Membranes in Reaction Coupling Separation Processes
Source: Materials (Basel). 2012 Oct 30;5(11):2101–36. doi: 10.3390/ma5112101 (PMC5448993; doi:10.3390/ma5112101)
Supplement: Supplementary File 1 [file materials-05-02101-s001.pdf]

Graphical Abstract

Potential Applications of Zeolite Membranes in Reaction Coupling Separation Processes

Michael O. Daramola [1,*], Elizabeth F. Aransiola [1,2] and Tunde V. Ojumu [2]

[1] Biochemical and Reactions Engineering Group, Department of Chemical Engineering, Obafemi Awolowo University, Ile-Ife 220005, Osun State, Nigeria; E-Mail: aransiola4@yahoo.com

[2] Department of Chemical Engineering, Cape Peninsula University of Technology, Cape Town 8000, South Africa; E-Mail: ojumut@cput.ac.za

* Author to whom correspondence should be addressed; E-Mail: kennydara@yahoo.com; Tel.: +31-707-439-216.

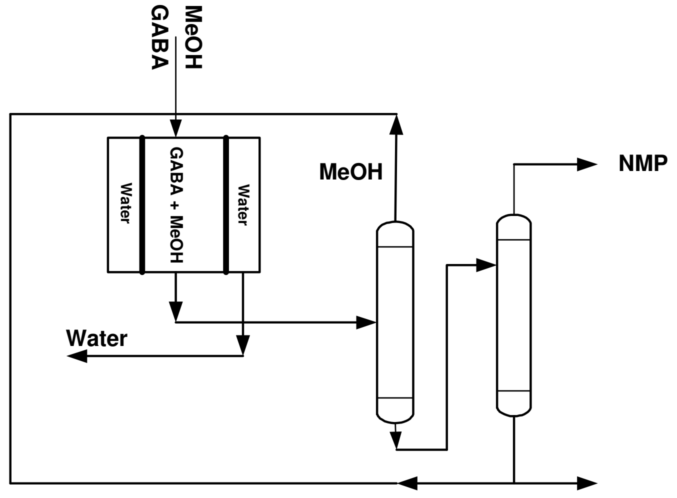

The figure shows an intensified unit involving the use of an inert zeolite catalytic membrane reactor (IZCMR) equipped with water selective membrane as the separation unit during the cyclization and methylation of γ-aminobutyric acid (GABA) with methanol (MeOH) to N-methlpyrrolidone (NMP) .
