# Supplementary material for: Potential Applications of Zeolite Membranes in Reaction Coupling Separation Processes
Source: Materials (Basel). 2012 Oct 30;5(11):2101–36. doi: 10.3390/ma5112101 (PMC5448993; doi:10.3390/ma5112101)
Supplement: Supplementary File 2 [file materials-05-02101-s002.pdf]

Materials **2013**, *6*, 1432-1433; doi:10.3390/ma6041432

OPEN ACCESS

materials

ISSN 1996-1944

www.mdpi.com/journal/materials

Correction

Correction: Daramola, M.O.; Aransiola, E.F.; Ojumu, T.V. Potential Applications of Zeolite Membranes in Reaction Coupling Separation Processes. *Materials* 2012, *5*, 2101-2136.

Michael O. Daramola [1,2,*], Elizabeth F. Aransiola [1,3] and Tunde V. Ojumu [3]

1 Biochemical and Reactions Engineering Group, Department of Chemical Engineering, Obafemi Awolowo University, Ile-Ife 220005, Osun State, Nigeria; E-Mail: aransiola4@yahoo.com
2 Catalysis Engineering Section, Delft University of Technology, Julianalaan 136, 2628 BL, Delft, The Netherlands
3 Department of Chemical Engineering, Cape Peninsula University of Technology, Cape Town 8000,South Africa; E-Mail: ojumut@cput.ac.za

* Author to whom correspondence should be addressed; E-Mail: kennydara@yahoo.com; Tel.: +31-707-439-216.

Received: 14 January 2013 / Accepted: 26 March 2013 / Published: 2 April 2013

Due to an oversight by the authors, Table 4 in the aforementioned review article [1] should have been referred to as obtained from reference [2]:

Khajavi, S. Separation of Process Water Using Hydroxy Sodalite Membranes. PhD Thesis, Delft University of Technology, Delft, The Netherlands, 2010.

Furthermore, an additional affiliation for the first author of this review is added as follows:

Catalysis Engineering Section, Delft University of Technology, Julianalaan 136, 2628 BL, Delft, The Netherlands

The authors apologize for any inconvenience this may have caused.

References

1. Daramola, M.O.; Aransiola, E.F.; Ojumu, T.V. Potential Applications of Zeolite Membranes in Reaction Coupling Separation Processes. *Materials***2012**, *5*, 2101-2136.

2. Khajavi, S. Separation of Process Water Using HydroxySodalite Membranes. PhD Thesis, Delft University of Technology, Delft, The Netherlands, 2010.

© 2013 by the authors; licensee MDPI, Basel, Switzerland. This article is an open access article distributed under the terms and conditions of the Creative Commons Attribution license (http://creativecommons.org/licenses/by/3.0/).
